# Definitive carbon ion re-irradiation with pencil beam scanning in the treatment of unresectable locally recurrent rectal cancer

**DOI:** 10.1093/jrr/rrad068

**Published:** 2023-09-20

**Authors:** Xin Cai, Ping Li, Jingfang Zhao, Weiwei Wang, Jingyi Cheng, Guangyuan Zhang, Sanjun Cai, Zhen Zhang, Guoliang Jiang, Qing Zhang, Zheng Wang

**Affiliations:** Department of Radiation Oncology, Shanghai Proton and Heavy Ion Center, Shanghai, China; Shanghai Key Laboratory of Radiation Oncology (20dz2261000), Shanghai, China; Shanghai Engineering Research Center of Proton and Heavy Ion Radiation Therapy, Shanghai, China; Department of Radiation Oncology, Shanghai Proton and Heavy Ion Center, Shanghai, China; Shanghai Key Laboratory of Radiation Oncology (20dz2261000), Shanghai, China; Shanghai Engineering Research Center of Proton and Heavy Ion Radiation Therapy, Shanghai, China; Shanghai Key Laboratory of Radiation Oncology (20dz2261000), Shanghai, China; Shanghai Engineering Research Center of Proton and Heavy Ion Radiation Therapy, Shanghai, China; Department of Medical Physics, Shanghai Proton and Heavy Ion Center, Shanghai, China; Shanghai Key Laboratory of Radiation Oncology (20dz2261000), Shanghai, China; Shanghai Engineering Research Center of Proton and Heavy Ion Radiation Therapy, Shanghai, China; Department of Medical Physics, Shanghai Proton and Heavy Ion Center, Shanghai, China; Shanghai Key Laboratory of Radiation Oncology (20dz2261000), Shanghai, China; Shanghai Engineering Research Center of Proton and Heavy Ion Radiation Therapy, Shanghai, China; Department of Nuclear Medicine, Shanghai Proton and Heavy Ion Center, Fudan University Cancer Hospital, Shanghai, China; Shanghai Key Laboratory of Radiation Oncology (20dz2261000), Shanghai, China; Shanghai Engineering Research Center of Proton and Heavy Ion Radiation Therapy, Shanghai, China; Department of Radiology, Shanghai Proton and Heavy Ion Center, Shanghai, China; Department of Colorectal Surgery, Fudan University Shanghai Cancer Center, Shanghai, China; Department of Radiation Oncology, Fudan University Shanghai Cancer Center, Shanghai, China; Department of Radiation Oncology, Shanghai Proton and Heavy Ion Center, Shanghai, China; Shanghai Key Laboratory of Radiation Oncology (20dz2261000), Shanghai, China; Shanghai Engineering Research Center of Proton and Heavy Ion Radiation Therapy, Shanghai, China; Department of Radiation Oncology, Shanghai Proton and Heavy Ion Center, Shanghai, China; Shanghai Key Laboratory of Radiation Oncology (20dz2261000), Shanghai, China; Shanghai Engineering Research Center of Proton and Heavy Ion Radiation Therapy, Shanghai, China; Department of Radiation Oncology, Shanghai Proton and Heavy Ion Center, Fudan University Cancer Hospital, Shanghai, China; Department of Radiation Oncology, Shanghai Proton and Heavy Ion Center, Shanghai, China; Shanghai Key Laboratory of Radiation Oncology (20dz2261000), Shanghai, China; Shanghai Engineering Research Center of Proton and Heavy Ion Radiation Therapy, Shanghai, China; Department of Radiation Oncology, Shanghai Proton and Heavy Ion Center, Fudan University Cancer Hospital, Shanghai, China

**Keywords:** carbon ion radiotherapy, pencil beam scanning, locally recurrent rectal cancer, re-irradiation

## Abstract

This study aimed to evaluate the oncological outcomes and safety of carbon ion re-irradiation with pencil beam scanning (PBS) delivery technique for previously irradiated and unresectable locally recurrent rectal cancer (LRRC). Between June 2017 and September 2021, 24 patients of unresectable LRRC with prior pelvic photon radiotherapy who underwent carbon ion re-irradiation at our institute were retrospectively analyzed. Carbon ion radiotherapy was delivered by raster scanning with a median relative biological effectiveness-weighted dose of 72 Gy in 20 fractions. Weekly CT reviews were carried out, and offline adaptive replanning was performed whenever required. The median follow-up duration was 23.8 months (range, 6.2–47.1 months). At the last follow-up, two patients had a local disease progression, and 11 patients developed distant metastases. The 1- and 2-year local control, progression-free survival and overall survival rates were 100 and 93.3%, 70.8 and 45.0% and 86.7 and 81.3%, respectively. There were no Grade 3 or higher acute toxicities observed. Three patients developed Grade 3 late toxicities, one each with gastrointestinal toxicity, skin reaction and pelvic infection. In conclusion, definitive carbon ion re-irradiation with PBS provided superior oncologic results with tolerable toxicities and may be served as a curative treatment strategy in unresectable LRRC.

## INTRODUCTION

Rectal cancer is one of the most common malignant tumors of the digestive system worldwide. Despite the great improvement of its clinical outcomes attributing to a multidisciplinary approach combining surgery, chemotherapy and radiotherapy, 5–10% of patients with locally advanced disease still develop pelvic recurrences after standard neoadjuvant radiation or chemoradiation followed by total mesorectal excision [1–3]. Moreover, the prognosis in this situation can be devastating, with a series of distressing symptoms including pain, bleeding, obstruction, fistula, discharge, urinary and fecal incontinence, which significantly affect the patients’ quality of life [4, 5].

Although salvage surgery with negative margin is recognized as the only potential curative treatment for locally recurrent rectal cancer (LRRC), unfortunately the majority of cases are judged to be unresectable [6]. Radiation therapy represents another option for local therapy, but it is normally administrated with neoadjuvant or palliative intention, because nearly all the recurrent lesions are located within the previous irradiation field and frequently in proximity to the radiosensitive critical organs of the pelvis. Thus, durable local tumor control would most probably not be achieved by moderate radiation doses, particularly considering a great proportion of hypoxic cells and cancer stem cells presented in recurrent tumors, which are commonly radioresistant. Albeit many efforts such as the modification of fractionation scheme and the use of advance technology have been made in the context of photon-based re-irradiation, the reported long-term tumor control rates for LRRC remain unsatisfactory [7, 8].

Compared to photon beams, carbon ions possess the characteristic Bragg peak of charged particles, and highly conformal dose distribution due to its narrow lateral and distal penumbras, which could spare the adjacent organs at risk (OARs) more effectively [9]. Furthermore, carbon ions are considered high linear energy transfer radiation that produces denser ionizations, leading to increased relative biological effectiveness (RBE) and a reduced oxygen enhancement ratio. This, in turn, potentially results in greater cell-killing effects, especially for radioresistant hypoxic tumor cells, which are common in the LRRC [10, 11]. To date, the Japanese studies reported the largest number of LRRC patients treated with either irradiation or re-irradiation with carbon ion radiotherapy (CIRT), showing encouraging results and acceptable toxicities [12]. However, their clinical data totally depended on the traditional beam delivery technique termed as passive scattering (PS). As a more advanced delivery technique, pencil beam scanning (PBS) provides intensity modulations in both lateral and depth direction to achieve a true 3D dose painting, and especially minimizes excessive dose to the normal tissues at the proximal side of the target compared to the PS [13]. Thus, its use may theoretically better spare the nearby OARs and further improve the toxicity profiles, which is of particular importance in the setting of definitive re-irradiation. Although two European centers previously published their clinical data of re-irradiation with PBS CIRT, it is unfortunate that only a dozen cases have been treated respectively [14, 15].

Therefore, the present study aimed to illustrate our oncological outcomes and safety of CIRT re-irradiation with PBS technique for unresectable LRRC, and add to the growing body of evidence on this issue.

## MATERIALS AND METHODS

### Study design and patients

This retrospective observational study enrolled LRRC patients who underwent re-irradiation using scanning carbon ion beams at Shanghai Proton and Heavy Ion Center. The inclusion criteria were as follows: (i) LRRC confirmed by biopsy, or clinically diagnosed using the criteria which have been previously described [16]; (ii) a history of radical surgery and pelvic photon radiotherapy for their rectal cancer; (iii) recurrent lesion judged as unresectable by surgeons in the colorectal cancer multidisciplinary therapy team of Fudan University Shanghai Cancer Center; and (iv) carbon ion re-irradiation performed for the recurrent disease. Patients were excluded if they (a) previously received more than one course of radiotherapy in the same site; (b) underwent re-irradiation within 1 year of last radiotherapy; or (c) had recurrent tumor invading the digestive tract and bladder. This study was reviewed and approved by the ethics committee of our center (Approval Number: 230407EXP-02), and all the patients signed an informed consent form before treatment initiation.

### Carbon ion radiotherapy

All patients were positioned supine or prone with immobilization devices and underwent CT simulation. Before simulation and every treatment session, patients were requested to empty the rectum using glycerin enema, as well as to have an empty or comfortable full bladder depending on the case, which may minimize their interfractional variations. The treatment planning CT images were acquired with a 2 mm slice thickness, and then were merged with the contrast-enhanced CT and MRI images to facilitate precise target delineation.

**Fig. 1:**
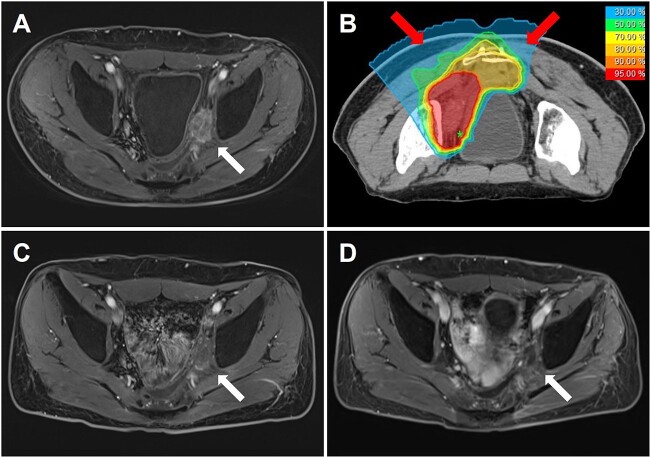
A 41-year-old male patient with pelvic side-wall recurrence of rectal cancer treated by carbon ion re-irradiation. (**A**) Baseline MRI with contrast enhancement before CIRT. (**B**) Dose distribution of the CIRT plan using 2 oblique posterior beams, with a prescription D_RBE_ of 75.6 Gy in 21 fractions. (**C**) Follow-up MRI 1 month after CIRT showing a responding tumor with significantly decreased enhancement. (**D**) Latest MRI taken 47 months after CIRT demonstrating no evidence of tumor recurrence.

The gross tumor volume (GTV) was defined as the macroscopically visible lesions on contrast-enhanced pelvic MRI, CT and PET imaging. The clinical target volume (CTV) was formed by expanding a 5–10 mm margin from the GTV, including all prechemotherapy GTV. In consideration of a high rate of subclinical tumor cells within the fibrotic area around the GTV, the fibrosis should also be irradiated [17]. The CTV_f_ was thus defined as CTV with inclusion of the entire fibrotic area. The planning target volume (PTV) and PTV_f_ were produced by adding margins of 3–5 mm around CTV and CTV_f_, respectively, for the setup error and beam range uncertainty. The goals for target volume coverage were that at least 95% of the PTV and 99% of the GTV had to be covered by 95% of the prescribed RBE-weighted dose (D_RBE_). In view of the re-irradiation situation, 80% of prescription dose was delivered to the PTV_f_, when available, for prophylactic purpose using the simultaneous integrated boost technique. The D_RBE_ constraints of the minimum dose received by the most exposed 2 cc volume (D_2cc_) for the intestine, rectum and bladder were 50, 60 and 60 Gy, respectively. Generally, two to three horizontal or 45° oblique beams with different couch positions were arranged. The prescription D_RBE_ was calculated by the local effect model version I (LEM-I) in the Syngo treatment planning software system. CIRT was administered by Siemens synchrotron with raster scanning, once daily, five times per week. The dose fractionation regimens were determined by the tumor location. Normally, a 15-fraction regimen were used, but for tumors that abutted or encased the adjacent peripheral nerves like the ischiatic nerve, a more moderate regimen of 20 fractions was adopted to minimize the late-onset radiation-induced nerve damage. In the case of tumors with large size or central necrosis, one more fraction was delivered when staying within the dose constraints for OARs. A representative case is shown in Fig. 1.

For patient position verification, a pair of orthogonal kilovoltage X-ray films was taken immediately before each treatment fraction to measure the setup errors, and corrections were made online by pelvic bone structure matching. For treatment adaptation, weekly repeated CT and potential replanning were performed to minimize the possibility of unacceptable geometric misses.

### Data collection

The baseline demographic and clinical characteristics, CIRT parameters, post-treatment imaging examinations including pelvic contrast-enhanced MRI, chest CT and PET scans and adverse events were retrospectively collected from the electronic medical record system.

Tumor response was evaluated by the Response Evaluation Criteria in Solid Tumors version 1.1 based on the consensus of radiation oncologists and at least one expert radiologist. Local control (LC) was defined as no evidence of the irradiated lesion progression based on MRI and/or CT images. The LC, progression-free survival (PFS) and overall survival (OS) were calculated from the first day of CIRT to the date of the events of interest. Patients without events were censored at the time of the last follow-up. Adverse events were assessed according to the National Cancer Institute Common Terminology Criteria for Adverse Events version 4.03. Those occurred within 90 days from CIRT initiation were defined as acute toxicities, while beyond 90 days, as the late ones.

### Statistical analysis

Descriptive statistics were used to explore the clinical characteristics of all the patients. The LC, PFS and OS rates were estimated using the Kaplan–Meier method. All statistical analyses were performed using SPSS software version 26.0 (IBM Corp., Armonk, NY, USA).

## RESULTS

### Baseline characteristics

From June 2017 to September 2021, 24 consecutive patients who met the eligibility criteria were included in this study. All the patients presented with single recurrent lesion. The median age of patients was 53 years (range, 41–74 years). The two most common relapse locations were either presacral region (41.7%) or pelvic sidewalls (37.5%). All patients had received prior pelvic photon radiotherapy for rectal cancer, and the median interval time between first course and re-irradiation was 45.8 months (range, 12.1–117.2 months). Nine of the 24 patients were initially irradiated in the neoadjuvant setting, 11 patients in the adjuvant setting and the remaining 4 for the pelvic recurrent diseases. The median prescribed D_RBE_ of CIRT was 72 Gy (range, 67.5–75.6 Gy) in 20 fractions (range, 15–21 fractions). The mean D_2cc_ of the intestine, rectum and bladder for re-irradiation was 33.8 (range, 0.4–50.0 Gy), 34.5 (range, 10.2–51.5 Gy) and 37.0 Gy (range, 2.7–60.0 Gy), respectively. All patients completed the scheduled CIRT without any interruption. Concurrent chemotherapy was not administrated. There were 1, 7 and 10 patients who received induction, adjuvant chemotherapy and the combination for re-irradiation, respectively. None of the patients had a salvage surgery after CIRT at the last follow-up visit. The patient and treatment characteristics are summarized in Table 1.

**Table 1:** Patient and treatment characteristics

Characteristics	No. of patients (%)
Median age (range), years	53 (41–74)
Gender	
Male	17 (70.8%)
Female	7 (29.2%)
KPS	
80	2 (8.3%)
90	14 (58.3%)
100	8 (33.3%)
Surgery of primary tumor	
Abdominoperineal resection	10 (41.7%)
Low anterior resection	12 (50.0%)
Hartmann’s resection	2 (8.3%)
Histology	
Adenocarcinoma	19 (79.2%)
Mucinous adenocarcinoma	4 (16.7%)
Signet ring cell carcinoma	1 (4.2%)
Sites of recurrence	
Presacral	10 (41.7%)
Pelvic side wall	9 (37.5%)
Perineal	4 (16.7%)
Perianastomosis	1 (4.2%)
Median GTV (range), cm^3^	57.1 (6.1–334.1)
Prior photon RT dose	
30 Gy in 10 fx	2 (8.3%)
45 Gy in 25 fx	4 (16.7%)
50 Gy in 25 fx	11 (45.8%)
50.4 Gy in 28 fx	5 (20.8%)
60 Gy in 30 fx	2 (8.3%)
Carbon ion re-irradiation D_RBE_	
67.5 Gy in 15 fx (BED_10_ = 97.9 Gy)	11 (45.8%)
72 Gy in 20 fx (BED_10_ = 97.9 Gy)	10 (41.7%)
75.6 Gy in 21 fx (BED_10_ = 102.8 Gy)	3 (12.5%)
Median re-irradiation interval (range), months	45.8 (12.1–117.2)
Chemotherapy	
Induction for re-irradiation	1 (4.2%)
Adjuvant for re-irradiation	7 (29.2%)
The combination	10 (41.7%)

KPS = Karnofsky performance score, GTV = gross tumor volume, RT = radiotherapy, D_RBE_ = RBE-weighted dose, fx = fractions, BED = biological effective dose

### Tumor control and survival

**Fig. 2:**
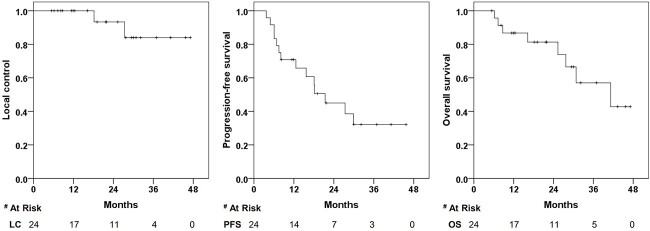
Kaplan–Meier estimates of LC, PFS and OS for all 24 patients.

The median follow-up duration was 23.8 months (range, 6.2–47.1 months) for all patients and 21.8 months (range, 7.1–47.1 months) for survivors. No patients were lost to follow-up and 16 patients were alive at the time of the last evaluation. One (4.2%) and 4 (16.7%) patients achieved complete response and partial response, respectively, while 17 (70.8%) had stable disease. Local disease progression was observed in two (8.3%) patients at 18.2 and 27.4 months after CIRT. All nine patients presented with pelvic pain prior to CIRT had varying degrees of symptom relief. In total, 11 patients developed distant metastases with the most common site being the lung. The tumor responses and distant failure patterns are detailed in Table 2. The 1- and 2-year LC rates were 100 and 93.3% (95% CI, 61.3–99.0%), respectively. The 1- and 2-year PFS rates were 70.8 (95% CI, 48.4–89.9%) and 45.0% (95% CI, 23.4–64.5%), respectively. The median OS time was 41.2 months (95% CI, 18.5–63.9%), with 1- and 2-year OS rates of 86.7% (95% CI, 64.3–95.5%) and 81.3% (95% CI, 57.3–92.6%), respectively. The Kaplan–Meier curves for LC, PFS and OS are shown in Fig. 2.

**Table 2:** Tumor responses and distant failure patterns

Parameters	No. of patients (%)
Response (*n* = 24)	
Complete response	1 (4.2%)
Partial response	4 (16.7%)
Stable disease	17 (70.8%)
Progressive disease	2 (8.3%)
Pain relief (*n* = 9)	
Complete relief	6 (66.7%)
Partial relief	3 (33.3%)
Distant metastases (*n* = 11)	
Lung	2 (18.2%)
Liver	1 (9.1%)
Lymph nodes	2 (18.2%)
Bone	1 (9.1%)
Lung and bone	2 (18.2%)
Lung and lymph nodes	1 (9.1%)
Others	2 (18.2%)

### Toxicity

Of the 24 patients, no Grade 3 or higher acute toxicity was observed. There were three patients who developed Grade 3 late toxicities, including one each with gastrointestinal (GI) toxicity, skin reaction and pelvic infection. The most frequent Grade 2 late toxicity was neuropathy, which was experienced by four (16.7%) patients. The adverse events that occurred after CIRT re-irradiation are described in Table 3.

**Table 3:** Acute and late toxicities

Toxicities	Acute						Late				
	G0	G1	G2	G3	G4–5		G0	G1	G2	G3	G4–5
Skin	22	1	1	0	0		20	2	1	1	0
GI tract	23	1	0	0	0		23	0	0	1	0
Neuropathy	24	0	0	0	0		18	2	4	0	0
Pelvic infection	24	0	0	0	0		22	0	1	1	0
Hematologic	16	6	2	0	0		24	0	0	0	0
Others	22	2	0	0	0		24	0	0	0	0

GI = gastrointestinal, G = grade

## DISCUSSION

The management of unresectable LRRC remains a big challenge especially for those patients with prior pelvic irradiation. Herein, we present our retrospective analysis of the efficacy and safety of carbon ion re-irradiation using PBS method for curative intention. The 2-year LC and OS rates were 93.3 and 81.3%, respectively. None developed Grade ≥ 3 acute toxicity, and 12.5% developed Grade 3 late toxicities which were acceptable and manageable. Together, the results were comparable to that of those resectable cases treated with complete resection, suggesting that dose-intensified carbon ion re-irradiation might be the optimal treatment strategy for patients with unresectable LRRC.

In the case of unresectable recurrence, besides systemic chemotherapy, radiotherapy still serves as a main strategy for local tumor control. However, an increasing proportion of patients have previously received pelvic radiotherapy for the primary tumor, which impedes the use of re-irradiation [18]. In addition, a great possibility of severe, sometimes catastrophic, morbidity resulting from disease progression makes more sense of repeated radiotherapy even with potential risk of toxicity. Previous reports on photon re-irradiation for unresectable LRRC commonly adopted the accelerated hyperfractionated schedules (1.2–1.5 Gy per fraction twice daily to total dose of 30–56.4 Gy), leveraging radiobiology to protect the adjacent normal tissues, but often achieving only a palliative effect due to the relatively low biological effective doses [19, 20]. A meta-analysis including 17 studies demonstrated that the pooled LC and OS rates at 1, 2 and 3 years were 72.0, 54.8 and 44.6%, and 63.5, 34.2 and 23.8%, respectively, along with the pooled Grade 3 or higher acute and late toxicity rates of 25.5% for patients treated with re-irradiation but not surgery [21]. More recently, the advanced photon radiation technique, stereotactic body radiation therapy, has been utilized in this setting as well. Smith *et al*. and Johnstone *et al*. successively reported the experiences from the United Kingdom centers for patients with small volume pelvic recurrences [22, 23]. Re-irradiation using an ultrahypofractionation scheme of 30 Gy in five fractions for these highly selective cases yielded a local relapse rate of 42.6% at the time of death or the last follow-up and a 2-year OS rate of 77%, with limited toxicities.

CIRT has been gradually investigated for LRRC patients either in the setting of first irradiation or re-irradiation in recent years. Japanese centers have summarized their experiences with PS beam delivery system in both settings. For the radiation-naïve cohort, treatment outcomes of over 200 patients were presented by three CIRT institutions in Japan. Using the prescribed D_RBE_ of 70.4–73.6 Gy in 16 fractions, the favorable data with LC rates of 77–88% and OS rates of 51–58% at 5 years were yielded, and also indicated that high total dose was positively correlated with clinical results [24–26]. While in the context of re-irradiation, Yamada *et al*. and Shiba *et al*. respectively reported 77 and 7 patients treated with the same 70.4–73.6 Gy in 16 fractions as non-re-irradiation studies for standard cases, and 57.6 Gy in 12 fractions for close-to-GI tract cases [27, 28]. Long-term outcomes of Yamada *et al*.’s study provided the 5-year infield and regional LC rates of 87 and 81%, respectively, and the 5-year OS rate of 38%, which were comparable to those of non-re-irradiation studies. As for treatment-related toxicity, both acute and late Grade 3 or higher complication rates in the re-irradiation setting seem to be slightly higher than those in the non-re-irradiation setting (acute, 0–10.4% vs 0–1.3%; late, 14.3–20.8% vs 1.7–7.1%).

Although the comparisons of dosimetric and clinical data between PBS and PS CIRT for LRRC are lacking, the PBS method is expected to be superior to PS one due to its active scanning manner in 3D by scanning magnets, enabling a more flexible dose distribution [29]. A dosimetric comparison between the two for locally advanced pancreatic cancer has been previously investigated, which showed that the PBS plans resulted in significantly lower doses to the stomach and duodenum regardless of the beam angles [30]. Certainly, it is noted that such advantages of PBS over PS would be disease- and case-dependent. Like us, the European CIRT centers employ PBS as the only treatment modality. The Heidelberg Ion Beam Therapy Center presented their findings of carbon ion re-irradiation with moderate D_RBE_ of 36–51 Gy in 12–17 fractions (68% of patients with 36 Gy in 12 fractions). Within a short median follow-up period of 7.8 months, the median survival was not reached, and up to 21.1% (4 out of 19) patients developed local relapse [14]. Afterwards, a study from the Italian National Center for Oncological Hadrontherapy (CANO) retrospectively analyzed 14 patients treated with 35–76.8 Gy in 15–20 fractions (a median of 60 Gy in 16 fractions), producing an OS rate of 52% and LC rate of 76.2% at 2 years [15]. Collectively, it seems that the not very high doses could contribute to their relatively poor treatment outcomes. In addition, no Grade 3 or higher acute and late toxicities were reported in the above two cohorts, which may be partly explained by the more advantageous beam delivery method. In the present study, definitive doses close to Japanese ones were delivered, which resulted in a tumor control rate comparable to Japanese data despite the well-known differences in RBE models used for D_RBE_ calculation between different carbon ion centers (microdosimetric kinetic model in Japanese facilities, and LEM-I in European ones and ours) [31], but it was certainly more favorable compared to those of European cohorts.

Regarding the toxicity from re-irradiation, late severe GI and peripheral nervous events like bowel perforation and bleeding, as well as neuropathy are the most concerned problems, which may be hard to salvage and even be fatal in extreme cases. A clinical comparison study of CIRT versus X-ray re-irradiation for LRRC was recently carried out, showing CIRT had lower rates of both severe late GI and genitourinary (GU) toxicity compared with X-ray radiotherapy, as expected [32]. For tumors that away from pelvic serial OARs such as the bowel and sciatic nerve, the use of carbon ion beams characterized by favorable dose distribution with reduced integral dose is enormously helpful in enabling ablative doses with large fraction sizes to be given while sparing these OARs. For tumors located very close to or within these OARs, the appropriate dose and fractionation should be chosen, rather than using advanced techniques, to minimize the complication risk of delivering definitive doses. Furthermore, more aggressive approaches have been proposed and clinically practiced to protect the adjacent OARs. A pre-CIRT spacer placement surgery which may physically separate the tumor and the bowel or bladdery with a Gore-Tex sheet has been reported in the Japanese and CANO’s cohorts. Although higher dose administration may be achieved to improve tumor eradication, its nonabsorbable nature brings a risk of spacer-related pelvic infection as well as subsequent GI or GU toxicity, especially in re-irradiation cases. Therefore, a novel and more ideal bioabsorbable polyglycolic acid spacer has been recently developed and preliminarily applied for abdominal and pelvic tumors. It can maintain 80% of its thickness for at least 8 weeks and will eventually disappear within 8 months after placement, thus decreasing the infection risk and avoiding a repeated surgery for its removal [33, 34]. Additionally, a colostomy before CIRT may reduce the exposure of the irradiated colorectal wall to passing feces, thereby potentially benefiting in alleviating toxicities [35].

The present study was hampered by several limitations. First, this was a retrospective analysis with a relatively short follow-up time. Additional long-term data are needed to confirm the current findings. Meanwhile, a prospective Phase II trial (clinicaltrials.gov identifier: NCT05807984) on this topic that would provide more reliable evidences is now recruiting patients at our center. Second, conclusions may be limited by the relatively small sample size as well as the heterogeneity in the presentation of LRRC, which attributes to the low incidence of this disease, the high expenditure of CIRT and also the single-center design of this study. Third, although evidence for the value of chemotherapy in LRRC is lacking, its application might affect the oncological outcomes by eradicating micrometastases and improving radiosensitivity. However, the statistical analyses could not be adjusted for it due to the small number of patients and widely varying regimens.

## CONCLUSION

Our study reported superior oncological results with tolerable toxicities from PBS CIRT for LRRC in the re-irradiation setting, which were comparable to those of surgery cohorts with clear resection margins. Definitive CIRT may be served as a curative treatment strategy in unresectable LRRC, and more data are warranted to verify the potential benefits of PBS technique on clinical outcomes, especially toxicity profiles.

## CONFLICT OF INTEREST

The authors declare that they have no conflicts of interest in relation to this study.

## FUNDING

This work was supported by a grant from Shanghai Pudong New Area Heath Commission (PW2022A-26).

## DATA AVAILABILITY

The data presented in this study are available on request from the corresponding author. The data are not publicly available due to privacy and ethical restriction.

## PRESENTATION AT A CONFERENCE

The data in this manuscript were not presented at any conference.
